# Development and Evaluation of a Combined Contagious Bovine Pleuropneumonia (CBPP) and Lumpy Skin Disease (LSD) Live Vaccine

**DOI:** 10.3390/v14020372

**Published:** 2022-02-11

**Authors:** Najete Safini, Soufiane Elmejdoub, Zahra Bamouh, Mohamed Jazouli, Jihane Hamdi, Zineb Boumart, Halima Rhazi, Khalid Omari Tadlaoui, Mehdi El Harrak

**Affiliations:** R&D Virology Laboratory, MCI Santé Animale, Mohammedia 28810, Morocco; s.belmejdoub@mci-santeanimale.com (S.E.); z.bamouh@mci-santeanimale.com (Z.B.); m.jazouli@mci-santeanimale.com (M.J.); j.hamdi@mci-santeanimale.com (J.H.); Z.boumart@mci-santeanimale.com (Z.B.); h.rhazi@mcisante-animale.com (H.R.); k.tadlaoui@mcisante-animale.com (K.O.T.); m.elharrak@mci-santeanimale.com (M.E.H.)

**Keywords:** *Mycoplasma mycoides* subsp. *mycoides* (*Mmm*), Lumpy skin disease (LSD), contagious bovine pleuropneumonia (CBPP), combined Mmm/LSDVvaccine

## Abstract

*Mycoplasma mycoides *subsp.* mycoides (Mmm)* is the causative agent of contagious bovine pleuropneumonia (CBPP). Lumpy skin disease (LSD) is a viral disease of cattle caused by lumpy skin disease virus (LSDV). LSD and CBPP are both transboundary diseases spreading in the same areas of Africa and Asia. A combination vaccine to control CBPP and LSD offers significant value to small-scale livestock keepers as a single administration. Access to a bivalent vaccine may improve vaccination rates for both pathogens. In the present study, we evaluated the LSDV/CBPP live combined vaccine by testing the generation of virus neutralizing antibodies, immunogenicity, and safety on target species. In-vitro assessment of the Mycoplasma effect on LSDV growth in cell culture was evaluated by infectious virus titration and qPCR during 3 serial passages, whereas in-vivo interference was assessed through the antibody response to vaccination. This combined Mmm/LSDV vaccine could be used to protect cattle against both diseases with a single vaccination in the endemic countries. There were no adverse reactions detected in this study and inoculated cattle produced high levels of specific antibodies starting from day 7 post-vaccination, suggesting that this combination vaccine is both safe and effective.

## 1. Background

*Mycoplasma mycoides *subsp.* mycoides* (*Mmm*) is the causative agent of contagious bovine pleuropneumonia (CBPP), a severe, contagious respiratory disease affecting cattle and characterized by anorexia, fever, and respiratory signs such as dyspnea, polypnea, cough and nasal discharges) [1]. Mmm belongs to the classical ‘*Mycoplasma mycoides* cluster [2]. CBPP remains 1 of the major health problems in cattle throughout Sub-Saharan Africa, resulting in losses of over $2 billion annually, according to the AU-IBAR [3,4,5]. Mortality rates can exceed 50% when the disease appears for the first time in herds [6].

Lumpy skin disease (LSD) is a viral disease of cattle caused by lumpy skin disease virus (LSDV). LSDV belongs to the genus *Capripoxvirus* of the family *Poxviridae* together with sheep pox (SPV) and goat pox (GTPV) virus [7]. LSDV contains a linear double-stranded DNA genome enveloped by a lipid bilayer [8]. LSDV transmission takes place by blood-feeding arthropods [9,10,11] The disease can manifest in different forms ranging from acute to unapparent, characterized by fever, lymphadenitis, skin nodules, lesions of the ocular, nasal, and oral mucous membranes, and can, in severe forms, sometimes lead to death—at around 10% mortality rate [12,13]. It can cause important economic losses within a cattle population, such as a drop in milk production, weight loss, skin damage and temporary or permanent sterility in both bulls and cows [14].

Both CBPP and LSD were eradicated from many countries mostly through stamping-out strategies or vaccination campaigns [15]. CBPP has been eradicated in several countries during Rinderpest control campaigns, the end of the programs has resulted in a reemergence and increase in the prevalence and incidence of CBPP in almost all Sub-Saharan countries [16]. It is now present in all countries south of the Sahara down to North Namibia, Zambia and South Tanzania [17,18,19]. There are few regions in Africa which are not even closer to 4O% of vaccine coverage [6,20]. While for LSD control, vaccination of all susceptible animals is the main pillar, supported by other control measures such as animal movement restriction and vector control [21].

Vaccination remains one of the most important tools for the control of both diseases. Many vaccines against CBPP have been described but, the one most used is produced with attenuated Mmm strains [22,23]. Currently, 2 strains are used for CBPP vaccination: T1/44 and T1sr. T1/44 strain is the most used on the African continent. This strain was sufficiently attenuated to protect cattle without post-vaccinal severe reaction; however, such reactions may still occur in the field, although this is rare. This vaccine strain can effectively protect herds when vaccinations are regularly performed and can be used for CBPP control on a wider scale [24,25,26]. For cattle vaccination against LSDV, there are primarily live attenuated vaccines based on attenuated strains of wild isolates passaged on cell culture. Three known vaccines for LSDV are largely used in the field: Neethling vaccine, Kenyan (KO-180) strain vaccine and sheeppox virus (SPPV), or goatpox virus (GTPV) Gorgan goat pox (GTP) vaccine [27]. The LSDV Neethling attenuated strain of South Africa origin has been attenuated through 61 passages on the chorio-allantoic membrane and used as a vaccine strain for decades in Africa, Middle East, and Europe [25,28,29]. This strain has advantages in protecting against the disease despite of reported post-vaccination reaction in vaccinated population [30]. The first inactivated vaccine has recently entered the market [31].

LSD and CBPP are both transboundary diseases spreading in the same area of Africa. However, LSD has rapidly spread through southeast Europe, the Balkans, Russia, and Kazakhstan since 2012. LSD is one of the emerging threats to Europe and Asia [30,32]. Recently in 2020, LSD outbreak was declared in Hong Kong [33]. LSD was caused by a different strain of LSDV than the LSD epidemic in the Middle East and Europe in 2015–2018 [33,34]. The development of combined Mmm/LSDV vaccine could improve vaccination coverage for cattle. Indeed, this association offers significant benefit for the small-scale livestock keepers in Sub-Saharan African pastoralists because this vaccine will be given in a single vaccination against both diseases. The objective of this study was to evaluate the Mmm/LSDV live combined vaccine that could be used to protect cattle against both diseases in one single shot in endemic countries. Access to a bivalent vaccine may improve vaccination rates for both pathogens.

## 2. Materials and Methods

### 2.1. Live Attenuated Pathogens Preparation

Mycoplasmas live attenuated pathogen was prepared after 2 passages of the working seed (WS) of Mmm T1/44 (strain obtained from CIRAD AF262936) [35]. The culture was made in 2 passages using 2L round bottom boiling flasks: for first passage (P1) preparation, 447.5 mL of Hayflick modified medium [36], were supplemented with 50 mL of equine serum (Wisent bioproducts, Quebec City, Canada) 10%, was inoculated with 25 mL of the WS at a seed ratio of 5% and incubated at 37 °C with same agitation (100 rpm) for 24 h. For the passage 2 preparation, 2 flasks of 450 mL from the same medium were inoculated with 10% of the P1 (50 mL) separately and incubated at 37 °C under the same agitation speed to have a backup culture if 1 is contaminated. After 24 h of incubation, the fermenter (Pierre Guerrin, France) was inoculated with 5% of the inoculum and fermentation was conducted at 37 °C, pH 7.2 with the same agitation speed (100 rpm) for 36 h.

LSDV live attenuated pathogens: The LSD Neethling strain [37] (ID: AF409138) [38] was propagated on primary lamb testis (LT) cells.

The preparation of primary LT cells is carried out according to Roger Adams method [39]. LT cells maintained in Dulbecco’s Modified Eagle’s Medium (DMEM) (from Thermo fisher scientific, Grand Island, NY, USA,) with 10% fetal bovine serum (FBS) (purchased from Wisent bioproducts). Primary cells allow for a better viral multiplication in comparison with the lineage cell. These cells were tested for their viral purity by detecting the presence of Bovine viral diarrhea/Border disease viruses, and mycoplasma contamination [40]. Contamination in tested cell lines was not found. For LSDV culture, the medium was removed and replaced by the inoculum at 0.01 multiplication of infection (MOI). After 1 h of incubation at 37 °C, the inoculum was removed, replaced with DMEM with 1% FBS and incubated during 5 days at 35 °C until the cytopathogenic effect (CPE) became apparent. The supernatant was then harvested, aliquoted and stored at −80 °C until use.

### 2.2. Bivalent Mmm/LSDV Vaccine Preparation

The bivalent vaccine was prepared by adding LSDV and Mmm T1/44 live live attenuated pathogens to an equal volume of a stabilizer (4% peptone, 8% sucrose and 2% glutamate) then freeze-dried in LSI lyodryer (Lyogroup, Telangana, India). The vaccine was formulated in an appropriate concentration of live attenuated pathogens according to respective titers to ensure Mmm 10^8^ CCU_50_/dose and LSDV 10^4.5^ TCID_50_/dose. Upon several previous internal trials: 10^4.5^ TCID_50_/dose were the right dose for LSDV, for Mmm 10^8^CCU_50_/dose (data not shown). The general titer we used for LSDV was between 3.5 and 4.5; for Mmm, the minimal required titer recommended by OIE is 10^7^CCU_50_/dose

### 2.3. Vaccine Titration

Before vaccination, the vaccine pellet was diluted by adding a phosphate buffered solution (PBS). Mmm T1/44 titration in the vaccine preparations was carried out by the standard method of microtitration and color change and calculation of the titer by using the Spearman–Karber formula and expressed (CCU_50_: color-changing units) [41]. LSDV titration was performed by 10-fold dilutions of the vaccine suspension in the medium with LT cell suspension and incubation 5 days at 37 °C. Infectious virus titration was calculated by the Reed-Muench method [42] and expressed by TCID_50_/dose.

### 2.4. In-Vitro Assessment of Mycoplasma Effect on Viral Growth in Cell Culture

The effect of Mmm on virus was tested by simultaneous inoculations of LSDV and Mmm on LT cells and follow-up of individual growth. Three passages were carried out followed by qPCR and titration of each pathogen at each passage level.

Mycoplasma effect on LSDV growth was assessed by coinfecting mixtures of Mmm/LSDV onto LT cells as following: for the first passage (P1), the growth medium was removed, and cells propagated in 25 cm^2^ flask were inoculated with LSDV then adsorbed for 45 min. Next, Mmm was inoculated onto cells with media. The infected cells were examined daily. When cytopathic effect of LSDV reached 80%, the cells were harvested and frozen. The cell and virus suspensions were thawed at room temperature and the virus suspension was used for the next passage. In passage P2, the infection of cells was performed with the P1 harvest without MOI calculation, the same was performed for P3. The infectivity titer and qPCR (Ct) were determined after each of the three passages.

### 2.5. Quantitative Real-Time PCR

Nucleic acid DNA was extracted from 200 μL of live attenuated pathogens suspension samples using Isolate II genomic DNA kit (from Bioline, Meridian bioscience, Newtown, OH, USA) and eluted in 100 μL of buffer according to the manufacturer’s instructions. The extracted DNA was eluted in a volume of 60 µL of buffer and stored at −20 °C until use.

A quantitative real-time PCR (qPCR) TaqMan assay targeting ORF074 gene coding for the intracellular mature virion envelope protein P32 within LSDV [43], was used to determine viral genetic loads in samples. Tests were performed in 96- well Optical Reaction Plates (Applied Biosystems, Thermo fisher Scientific, Sainte Geneviève des Bois, France), contained 10 μL Luna Universal probe QPCR Master Mix (New England Biolabs, France), 1 µL of each capripoxvirus primer (10 µM), 0.5 µL of capripoxvirus probe (10 µM) [44], 4 μL of DNA template (25 ng/µL) and nuclease-free water (New England biolabs, France) to 20 μL. PCR was performed on the Quant Studio1 System (QS1 Applied Biosystems) using the following amplification program: 95 °C for 10 min; 45 cycles of 95 °C for 15 s and 60 °C for 1 min. For Mmm: PCR was performed using the same amplification reagent Luna (NEB Biolabs, and QS1 equipment. 20 μL reactions containing, 1.8 µL of each TaqMan primer (10 µM) and 0.6 µL TaqMan probe (10 µM) specific to the lipoprotein gene lppQ of Mmm as described by [45].

The amplification conditions were 95 °C during 5 min; and 40 cycles of 95 °C during 15 s and 54 °C for 1 min.

Primers sequences:

PPCB T1/44 Fwd-CTAGAACTGAGGTTTTAGTAATTGGTTATGA

Rev-CACGCTCTAGACTAATAATTTCTTCTGGTA

PPCB-probe-Fam-AAAAATTTCTGGGTTTGCTCAA-Tamra

SPV-LSD-GPV Fwd-AAA ACG GTA TAT GGA ATA GAG TTG GAA

Rev-AAA TGA AAC CAA TGG ATG GGA TA

FAM-TGG CTC ATA GAT TTC CT-MGB

Data were analyzed using the QS1 System software. Results were generated by determination of the threshold cycle (Ct).

### 2.6. Vaccine Safety and Immunogenicity Evaluation

Safety and serological response to the combined vaccine were tested on a group of 48 cattle. Holstein breed of 4–6 months old were housed in BSL3 facility. Cattle were first tested negative for the presence of LSDV antibodies using virus neutralization technique (VNT) and antibodies against Mmm using ELISA test.

The experiments were carried out in accordance with ARRIVE guidelines (https://arriveguidelines.org/ (accessed on 14 June 2021) and handling of experimental animals as described in a protocol approved by “The multi-chemical industry: MCI Santé Animale Ethic Committee for Animal Experiment Protocol number MCI-R70A1076 approved in September 2018”. The experimental works have been conducted in accordance with relevant national legislation on the use of animals for research and following code of practice for the housing and care of animals used in scientific procedures [46].

Cattle were fed a complete balanced diet and water ad libitum during acclimatization and experimental periods. Cattle were randomly selected and were divided into two groups, including those to be vaccinated and those to serve as controls. A total of 44 animals were vaccinated by subcutaneous route at day 0 and each animal received 2 mL of the vaccine preparation with a dose of 10^4.5^TCID_50_/dose of LSDV and 10^8^ CCU_50_/dose of Mmm. Four cattle were used as unvaccinated control.

All animals were observed and examined daily for clinical signs. Clinical scoring was based on general behavior, food uptake, abnormal local and systemic reactions, including inflammation at the injection site and nasal discharge. Rectal temperature was recorded for each animal 2 days prior to vaccination and at the time of vaccination and daily up to 14 DPV. Animals were monitored three months after the vaccination for antibody response. Blood samples were obtained on days 0, 7, 14, 21, 28, 35, 42, 56 and 90 DPV from both the control and vaccinated animals. All samples were tested for LSDV antibody by the VNT method as described in the OIE Terrestrial Manual and by ELISA for Mmm antibody [47,48]. All procedures were followed in accordance with the international guidelines for care and handling of experimental animals and approved by the internal Ethic Committee for Animal Experiment. Animals were observed daily during 14 days after vaccination for the following parameter scoring.

To preserve the trial’s objectivity, animal carers or investigators were blinded to the vaccine type and dose. Roles and responsibilities were prespecified. Clinical signs were scored as described in Table 1; a total cumulative score of assessed signs per animal and group per day were calculated. Sera were stored at −20 °C until analysis. At the end of the experiment, cattle were euthanized using humane slaughter.

### 2.7. Serological Testing

Nasal and buccal swabs were collected at days 3, 5, 7, 9 and 12 DPV for analysis by qPCR. Sera were collected weekly and analyzed for LSDV by Sero-neutralization and Interferon gamma (IFN-γ) assays, while for Mmm the serological response was assessed by ELISA (IDEXX CBPP Ab Test).

Virus neutralization (VNT) for LSDV was conducted as described by the OIE Manual (Chapters 2.7.11 and 2.7.14). This test is based on a serial ¼ dilutions of heat inactivated sera and a set amount of infectious virus titer (100 TCID_50_). The neutralizing antibody titer was calculated in accordance with Reed and Muench method [42].

ELISA against Mmm: Serological analysis for Mmm response was performed by IDEXX c-ELISA kit acquired from CIRAD (Montpellier, France) according to the manufacturer’s instructions and results were read at a wavelength of 450 nm.

### 2.8. Cell-Mediated Immunity

To assess the cell mediated immune response on animals vaccinated with LSDV inactivated vaccine, the Interferon Gamma (IFN-γ) levels upon stimulation of the heparin blood were examined using the Bovigam TB kit (purchased from Applied Biosystems, Thermo Fisher Scientific, France). Blood samples were incubated overnight with LSD virus, the derivative of a “pokeweed” protein used as a positive control and a blank (PBS) to stimulate lymphocytes. Then, the IFN-γ present in the plasma supernatant of each blood sample was determined using a sandwich ELISA.

## 3. Results

### 3.1. In-Vitro Mmm/LSDV Effect Assessment

**Figure 1:**
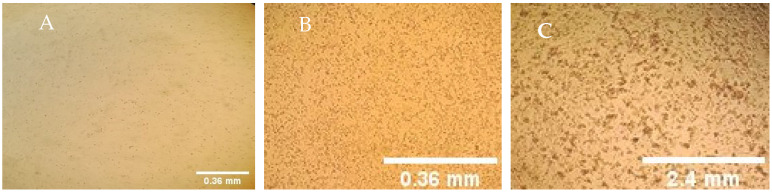
CPE of LSDV separated and in co-infection with Mmm on LT cell culture (×400). (**A**) LT infected with Mmm alone. (**B**) LSDV CPE effect, (**C**) Mmm/LSDV co-culturing CPE effect on LT cells.

The lamb Testis cells sensitivity to LSD virus cocultured with Mmm strain was evaluated by the appearance of LSDV cytopathic effect and the level of virus accumulation. When separately inoculated, LSDV induced round cells named Guarnieri bodies (Figure 1B). These bodies are slow to spread on the cell monolayer, whereas, Mmm mycoplasma had no visible effect on LT cells after 4 days incubation (Figure 1A); however, pox virus CPE was noticed 2–3 days post-infection (DPI). In co-cultivation of LSDV with Mmm strain, we observed foci of LSDV that appear on 2 DPI and was not interrupted by Mmm growth (Figure 1C).

Three passages were carried out, infectious virus titration and qPCR of each pathogen were performed at each passage level. Mycoplasma growth showed the absence of mycoplasma negative effect on viral growth in cell culture when tested by simultaneous inoculations of the 2 pathogens on cells and follow-up of individual growth (Table 2). Slight drop of Ct of LSDV in monovalent compared to combined suspension (16.6 in Passage 1 vs. 15.6 in P 3) neither of the LSDV titer when co-cultured with Mycoplasma 6.9 in P1 vs. 6.2 in P3 TCID_50_/mL.

### 3.2. Vaccine Testing on Cattle

After vaccination, 24 cattle (54.5%) presented moderate hyperthermia between 2 and 3 DPV. Body temperatures of unvaccinated cattle remained normal. All animals, including vaccinated and unvaccinated cattle, remained in good health. Clinical scoring was 0.9 for vaccinated animals. Unvaccinated cattle did not show any clinical signs.

No cases of Neethling disease such as were observed in all vaccinated cattle, during the 14 days post vaccination observation period. Local inflammatory reactions of 2 × 2 cm^2^ of diameter were observed on the inoculation site in four cattle (9.1%) of vaccinated group started at 4 DPV.

The possibility that combined Mmm/LSDV vaccine could spread among animals was addressed by housing vaccinated animals with unvaccinated animals. Assay of blood samples showed that all samples from unvaccinated cattle were negatives for LSDV and Mmm antibodies, indicating that vaccine did not spread from the vaccinated animals to unvaccinated animals (negative qPCR Cts for Mmm and for LSDV of the nasal and buccal swabs (data not shown)).

### 3.3. Serological Responses

**Figure 2:**
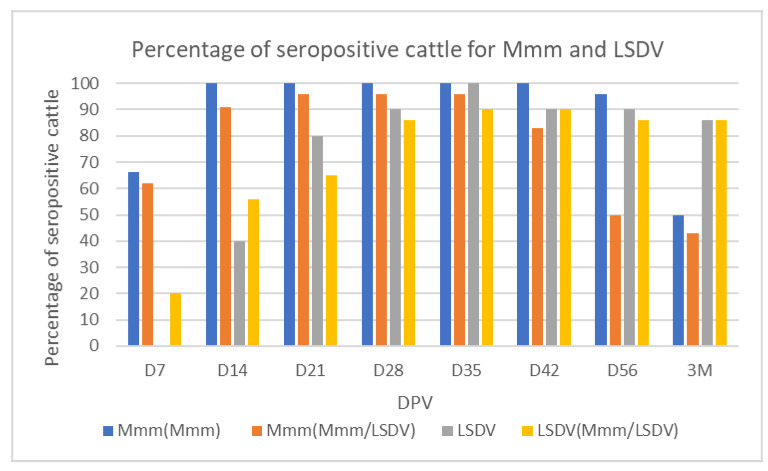
Percentage of seropositive cattle for Mmm and LSDV vaccinated with Mmm and LSDV monovalent vaccines vs. Mmm/LSDV combined vaccines monitored 3 months. D: Days M: Months.

Serological responses of the combined Mmm/LSDV vaccine in cattle was evaluated by vaccinating 24 naïve cattle, with 10^8^ CCU_50_/dose and 10^4.5^ TCID_50_/dose for Mmm and LSDV, respectively. Cattle vaccinated with combined Mmm/LSDV vaccine developed detectable antibody anti-Mmm by day 7 PV; 60% of tested cattle seroconverted for Mmm in the combined vaccine group vs. 66% for the monovalent group. All vaccinated cattle (100%) seroconverted at 14 DPV in the monovalent vaccine group and at 21 DPV in the combined vaccine group (Figure 2).

Neutralizing antibodies against LSDV were detected in the vaccinated animals with combined vaccine at 7 DPV (20%), while 40% of the monovalent vaccinated group seroconverted only at 14 DPV. The maximum response is obtained on 35 DPV (100% & 90% seroconversion for LSDV monovalent and Mmm/LSDV, respectively) (Figure 2).

**Figure 3:**
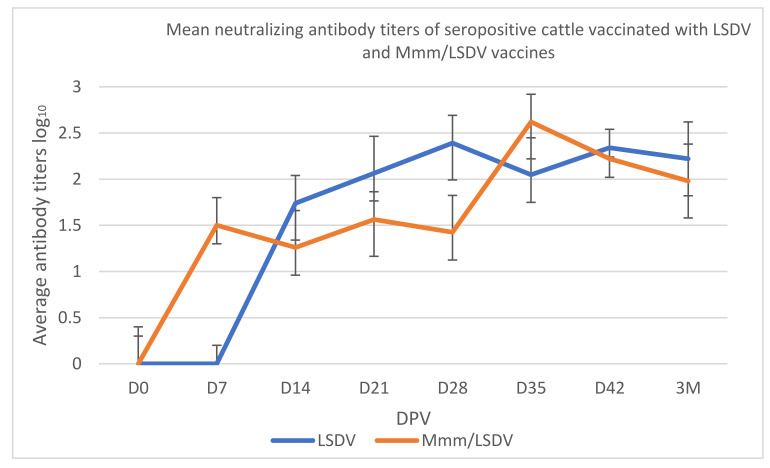
Mean neutralizing antibody titers of seropositive cattle vaccinated with LSDV and Mmm/LSDV vaccines monitored 3 months. Data shown are means of at least three neutralization experiments. D: Days M: Months.

The neutralizing antibody response of cattle to LSDV slightly differed in comparison to the response to a combination of Mmm/LSDV, with the response to combined viruses being shifted (2.62 log_10_ at 35 DPV) than the response to LSDV alone (2.39 log_10_ at 28 DPV) (Figure 3). There was an LSDV antibody response to Mmm/LSDV at 7 DPV, while for the monovalent, the immunological response was at 14 DPV. 

The cellular immunity was evaluated for LSDV using the quantification of interferon gamma in both monovalent LSDV and combined Mmm/LSDV vaccinated cattle. There was a slight decrease in positive cattle: 10% for IFN-γ vaccinated with combined Mmm/LSDV vs. LSDV vaccinated group. The cattle vaccinated with LSDV and Mmm/LSDV shows 88% of animals reacted positive to IFN-γ with LSDV while 71% in combined Mmm/LSDV vaccine (Table 3).

## 4. Discussion

LSDV and Mmm continue to spread despite effort of vaccination regularly conducted by several countries. Mitigation efforts to curb the spread of LSDV and Mmm are hindered by low vaccination coverage due to low efficacy of Mmm available vaccines, large pastoral margin, poor infrastructures to reach nomadic herds and availability of an efficient multivalent vaccine [25,49]. Vaccination against both Mmm and LSDV could be an interesting tool to control both diseases in cattle.

To our knowledge, this is the first work where both Mmm and LSDV are combined in one shot live vaccine. The main objective of the present study was to assess the effect of single or combined Mmm/LSDV vaccination in-vitro and in-vivo testing on cattle. Thus, the study design enables us to investigate the level of seroconversion to Mmm tested by ELISA and neutralizing antibodies against LSDV as well as cellular immune response. When cell culture growth of LSDV in Mmm infected cells, apparently the latter does not affect the infectious virus titration of LSDV. Even if cells co-infection with Mmm and LSDV has not been previously reported, and although several authors noticed that cells contaminated with mycoplasma loose sensitivity to viruses.

Mycoplasmas can affect virus propagation [50], and the effect is dependent upon the type of the mycoplasma strain. While some mycoplasmas have no effect on virus yield can increase virus yields with arginine-utilizing mycoplasmas can decrease the titer of vaccinia virus [51]. It has been also cited that contamination of chick embryo cells by *Mycoplasma gallisepticum* markedly affects the in-vitro multiplication of fowlpox virus and modifies the plaque morphology; this mycoplasma decreases the in-vivo infectivity of fowlpox virus for day-old chicks [52,53]. On the other hand, the chronic infection of hamster embryo fibroblasts by M. arginine and M. hyorhinis significantly increased the yield of two arboviruses, vesicular stomatitis (Indiana strain) and Semliki Forest viruses [52]. This enhancement resulted from a non-arginine dependent depletion of interferon production induced in infected cells by mycoplasmas.

The combined vaccine is safe when tested on target species, although it shows comparatively minimal adverse effects to monovalent vaccines. All animals stayed healthy through the experiments, although vaccination with Neethling strain has been documented to induce an LSD-like lesions at a low percentage for an unknown reason. An incidence of 0.38% (9/2356) of Neethling associated disease was observed among Neethling vaccinated cows [30]. CBPP vaccination has also been reported that the T1/44 vaccine may cause severe local reactions in cattle if there is any break in annual vaccinations [54]. Combination of both strains does not seem to increase residual virulence of the attenuated strains in our study.

In terms of immunogenicity, combined vaccination was able to cumulate the positive effects of each vaccine without any negative interference between strains. In fact, the serological monitoring using ELISA for Mmm and VNT for LSDV did not show any negative interference between vaccines when combined. Regarding Mmm serological response, we showed that the seroconversion of animals remains the same (100% vs. 90%) after vaccination compared to monovalent vaccinated animals (Figure 2). Same for LSDV humoral and cellular response; no difference was noticed between the two groups in term of kinetics or strength of the humoral immune response to LSDV neither for IFN-γ expression levels (Figure 3 and Table 3). Jores et al., assessed IFN-γ release after stimulation of peripheral blood mononuclear cells (PBMC) from experimentally infected cattle with Mmm. No correlation between IFN-γ release of animals and disease was noticed after stimulation of PBMC [55]. That could explain the absence of any interference between LSDV and Mmm (Table 3). This bivalent vaccine combining mycoplasma and a virus efficiently pooled the efficacy of each single CBPP and LSD vaccination. Very recently, the efficacy of the Neethling strain MCI vaccine in comparison with the other commercially available ones was experimentally evaluated using a challenge trial by the experts of the European Union LSD reference laboratory at Sciensano in Belgium and proven to be protective and showed the best serological response [56]. Other successful combination virus and mycoplasma was also reported. A combined vaccine associating two major pathogens involved in Porcine respiratory disease complex are *Mycoplasma hyopneumoniae* (Mhp) and porcine reproductive and respiratory syndrome virus (PRRSV). In their research, Bourry et al., reveal that combining Mhp and PRRSV MLV1 vaccines did not induce any negative interference which would reduce the efficacy of each individual vaccine and that combined vaccination is more efficient than the single ones for controlling a dual Mhp/EU-PRRSV infection in pigs [57]. Another swine combined vaccine, this new ready to use combination vaccine is shown to be safe and efficacious against Porcine CircoVirus type 2 and Mhp single and combined infections [58].

As vaccine coverage in Central, Eastern and Western Africa has declined since the closure of the Pan-African Rinderpest Campaign (PARC) in 1999 since during PARC vaccines against both rinderpest and CBPP were routinely administered to cattle in Central, Eastern and Western Africa [16]. The decision to vaccinate should be considering the economic effects of vaccination, the choice and availability of vaccine, the timing and spatial extent of vaccination [59]. Hence, Access to a bivalent vaccine may improve vaccination rates for both Mmm and LSDV.

## 5. Conclusions

The results of this study show that the combination, live Mmm/LSDV vaccine elicit a detectable antibody response similar to the monovalent vaccinations with minimal adverse reactions in cattle. Our results demonstrated that the Mmm/LSDV Neethling strain combined vaccine compared to their corresponding monovalent did not interfere in animal immunological response. However, a challenge study in cattle is needed before proceeding with widespread use. Access to a bivalent vaccine may improve vaccination rates for both pathogens.

## Figures and Tables

**Table 1:** Scoring of recorded clinical signs.

Criteria	0	1	2	3	4	5
General behavior	Normal	Weakness	Apathy	-	-	-
Appetite	Normal	Weak	Anorexia	-	-	-
Hyperthermia	Normal	+1 °C	>+1 °C	-	-	-
Local reactions	Absence	<2 cm	>2 cm	-	-	-
Respiratory symptoms	Normal	Discharge/cough	Dyspnea/polypnea	-	-	-
Neethling disease	None	1 to 2 nodules in one area	Up to 5 nodules in one area	>5 nodules in two areas	>5 nodules in three areas	Generalized nodules

**Table 2:** Means of qPCR cycle threshold (Ct) and infectious titrations obtained results after infection of LT cells with LSDV and Mmm and coinfection with Mmm and LSDV on the other hand. Infectious virus titration of LSDV in TCID_50_/_mL_ and CCU/mL for Mmm. SD: Standard Deviation (n = 3). P: passages on LT cells.

	Tested Pathogen	qPCR (Ct)(±SD)			Titer (±SD)		
		P1	P2	P3	P1	P2	P3
LSDV	LSDV	14.9 ± 0.6	14.3 ± 0.7	14.2 ± 0.5	6.8 ± 0.1	6.6 ± 0.4	6.3 ± 0.3
Mmm/LSDV	LSDV	16.6 ± 0.8	13.8 ± 0.6	15.6 ± 0.7	6.9 ± 0.3	6.5 ± 0.3	6.2 ± 0.5
	Mmm	24.5 ± 0.5	20.7 ± 0.5	18.9 ± 0.8	-	-	-

**Table 3:** Interferon Gamma Bovigam assay assessment in cattle vaccinated with LSDV and Mmm/LSDV.

Vaccine	LSDV Titer TCID_50_/Dose	Positive	% Positive Animals
LSDV	4.5	8/9	88
Mmm/LSDV	4.5	17/24	71

## Data Availability

The datasets used and/or analyzed during the current study are available from the corresponding author on reasonable request. All recorded raw data are archived in MCI Santé Animale.
